# Mechanisms and implications of vascular-homing CD8 T cells in atherosclerosis

**DOI:** 10.1038/s44325-025-00056-8

**Published:** 2025-05-28

**Authors:** Xi Su, Katelyn A. O’Hare, Michael L. Freeman

**Affiliations:** 1 Rustbelt Center for AIDS Research, Division of Infectious Diseases and HIV Medicine, Department of Medicine, Case Western Reserve University School of Medicine, Cleveland, OH USA; 2 Center for Global Health and Diseases, Department of Pathology, Case Western Reserve University School of Medicine, Cleveland, OH USA; 3 Present Address: Institute for Immunology, Department of Medicine, University of Pennsylvania Perelman School of Medicine, Philadelphia, PA USA

**Keywords:** Atherosclerosis, Immunology

## Abstract

CD8 T cells likely contribute to atherosclerosis. Here, we review the relationship of vascular-homing CD8 T cells to atherosclerotic cardiovascular disease, with discussions of atherogenic and atheroprotective CD8 T cell subsets, encompassing their origin, activation, antigen-specificity, trafficking, and functionality. Furthermore, we explore factors that promote CD8 T cell vascular-homing phenotypes, such as infections and inflammation, and describe innovative therapeutic strategies targeting vascular-homing CD8 T cells in people with atherosclerosis.

## Introduction

**Fig. 1:**
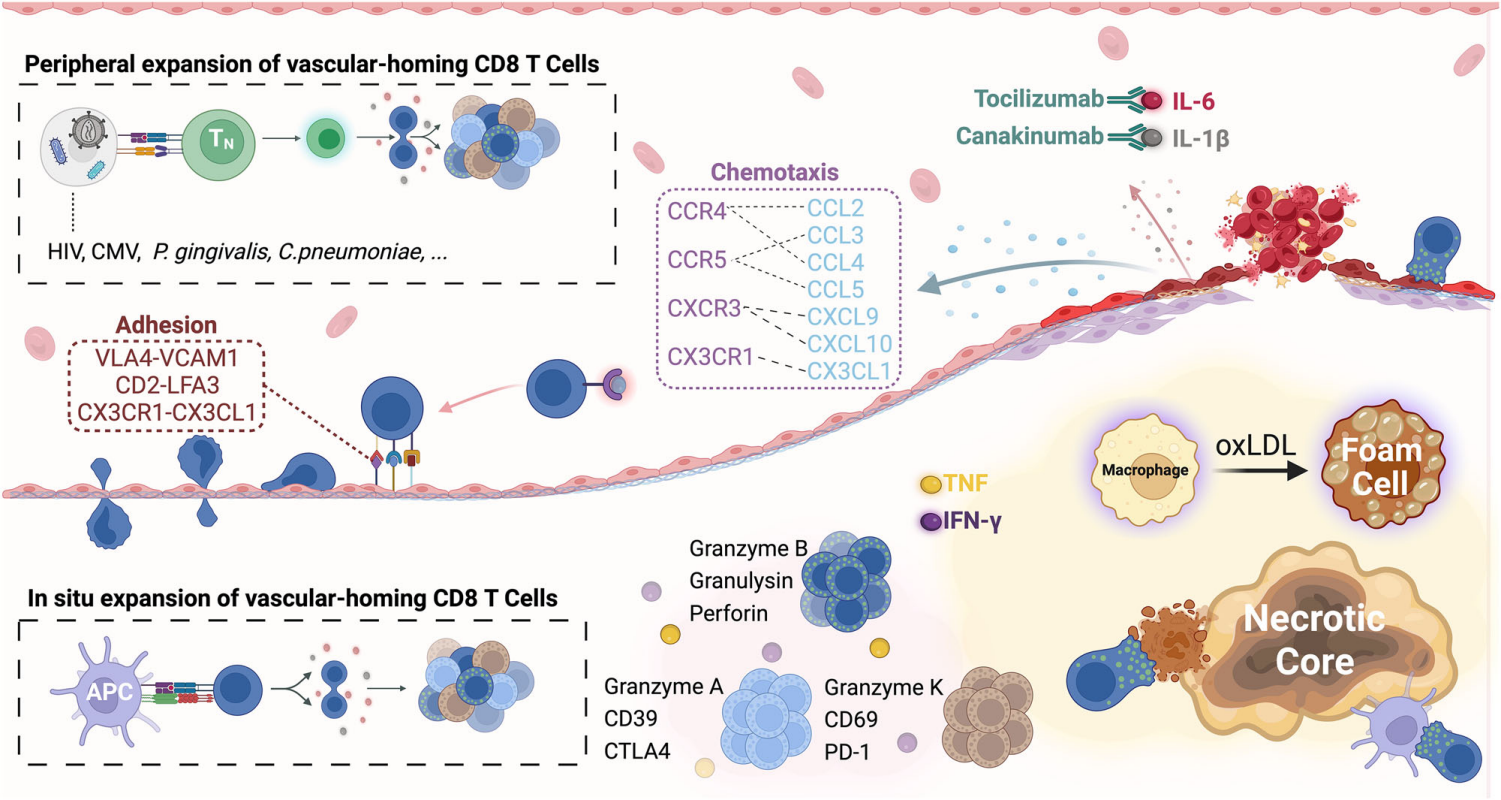
Overview of key concepts. Schematic illustrating the role of CD8 T cells within atherosclerotic plaques including cytokine and chemokine signaling, and antigen-specific T cell activation.

Atherosclerotic cardiovascular diseases (ASCVD) are the leading cause of mortality worldwide^1^. Hypercholesterolemia has been well established as the pivotal etiology for atherosclerosis, where the accumulation of oxidized low-density lipoprotein (oxLDL) in the artery wall initiates an inflammatory cascade that triggers the influx of innate and adaptive immune cells into the nascent plaque^2^ (Fig. 1). Plaque buildup restricts blood flow, increasing the risk of severe cardiovascular events, and plaque rupture can cause additional life-threatening complications such as thromboembolism. Despite the widespread use of statins to mitigate lipid levels, atherosclerosis-related mortality has continued to rise^3,4^, highlighting the need for a more comprehensive treatment approach. The significance of inflammation in atherosclerosis has been increasingly acknowledged, particularly in chronic inflammatory conditions such as rheumatoid arthritis (RA), and latent cytomegalovirus (CMV) and human immunodeficiency virus (HIV) infections, in which an increased burden of atherosclerosis is observed independently of lipid level^5,6^. This role of inflammation in atherosclerosis is further supported by evidence that targeting the proinflammatory cytokine interleukin-1β (IL-1β) with canakinumab reduced recurrent cardiovascular events by 15% in patients with established atherosclerosis, irrespective of lipid levels^7^.

The inflammatory pathogenesis of atherosclerosis begins with the infiltration and activation of monocytes, which transform into lipid-laden foam cells upon uptake of oxLDL, followed by recruitment of adaptive immune cells through proinflammatory signals including tumor necrosis factor (TNF), IL-1β, and IL-6^8^. CD8 T lymphocytes, a major component of the adaptive immune system, are major histocompatibility complex type I (MHC-I)-restricted cells with proliferative, effector cytokine production, and cytotoxic abilities. CD8 T cells are among the first immune cells to infiltrate atherosclerosis lesions and represent one of the largest portions of infiltrating immune cells, particularly in the later stages of atherosclerosis^9,10^. CD8 T cells in atherosclerotic lesions present with more activated phenotypes than those cells in circulation, and their abundance is strongly linked to severe ASCVD outcomes^9,11^. Studies of their role in ASCVD utilizing mouse models have offered some functional insight but with conflicting findings. Investigations in humans have been limited to primarily analyzing cells in circulation rather than those within the atherosclerotic plaque. However, recent investigations utilizing single-cell analysis dedicated to human atherosclerotic plaques, such as flow cytometry, RNA sequencing, and mass-cytometry, have revealed novel aspects of vascular-homing CD8 T cells and their relationship to the plaque microenvironment. This review article seeks to offer an inclusive discussion of these emerging aspects, highlighting the inflammatory nature of atherosclerosis and advancing insight into vascular-homing CD8 T cell subsets regarding their origin and function in atherosclerosis.

## Atherogenic and atheroprotective functions and mechanisms

CD8 T cells specific for hypercholesterolemia-derived self-antigens, which are also found in atherosclerotic lesions in mouse models, have been detected at frequencies positively associated with hypercholesterolemia and disease progression, suggesting a potential role in vascular homing and disease progression^12,13^. Study of vascular homing CD8 T cells of human atherosclerosis plaque revealed heightened activation and antigen-experienced phenotypes^10^. Surface markers of T cell memory and cellular activation, including CD25, CD38, CD45RO, CD69, and HLA-DR, are elevated on CD8 T cells in atherosclerotic plaque compared to those in circulation^10,14–17^. Upregulation of CD69 holds functional significance for the retention of vascular-homing CD8 T cells within the plaque by antagonizing sphingosine-1-phosphate (S1P)-mediated egress^18^. However, whether vascular-homing CD8 T cells are atherogenic or atheroprotective is still unclear.

In nonatherosclerotic arteries, a modest population of CD8 T cells can be found at the intima or adventitia of both humans and mice, indicating a baseline level of immunosurveillance^9,19^, and early experimental models with varying degrees of immunodeficiency at birth suggested an insignificant or even protective role of CD8 T cells in atherosclerosis^20,21^. Subsequent research employing more sophisticated models have uncovered both atherogenic and atheroprotective roles of CD8 T cells. Depletion of CD8 T cells in ApoE-/- and LDL-receptor (Ldlr)−/− mice by monoclonal antibodies prior to disease development showed reduced lesions with diminished accumulation of lipids and macrophages and a reduced necrotic core^22,23^. Similarly, mice lacking CD8 T cells showed reduced atherosclerosis in a model of *Chlamydia pneumoniae* infection^24^. In these studies, CD8 T cells were absent prior to inducing atherosclerosis, suggesting CD8 T cells are necessary for initial lesion formation. In contrast, antibody depletion of CD8 T cells in Ldlr−/− mice with advanced atherosclerosis resulted in plaque destabilization, characterized by an increase in macrophage presence, a larger necrotic core, and a less pro-calcifying smooth muscle cell phenotype, suggesting that CD8 T cells in advanced lesions may be atheroprotective by promoting plaque stability^25,26^.

This duality highlights that the distinct functions and pathologic implications of vascular-homing CD8 T cells are likely contingent on the context or target within the complex lesional microenvironment (Fig. 1). Recent applications of high dimensional immunophenotyping techniques to human atherosclerotic plaques have revealed novel aspects of phenotypic diversity with transcriptomic resolution and functional implications, establishing a close connection between the pathogenesis of atherosclerosis and phenotype and frequency of vascular-homing CD8 T cells.

CD8 T cells can be found throughout human atherosclerotic plaques but are primarily localized at the shoulder and fibrous cap region, exhibiting a broad range of immunological states. Among these, effector memory (T_EM_) cells are the most abundant subtype. CD8 T_EM_ are thought to contribute to lesional cell death and necrotic core formation through cytotoxic molecules, including perforin, granulysin, and granzymes, and proinflammatory cytokines including TNF and IFNγ^10^ (Fig. 1). In lymphocyte-deficient ApoE−/− Rag2−/− mice, adoptive transfer of CD8 T cells deficient in perforin, granzyme B (GzmB), or TNF, but not IFNγ, led to reduced atherosclerosis compared to the disease that develops with adoptive transfer of wild-type CD8 T cells, despite similar cellular infiltration into the lesion^22^. In addition to reduced macrophage and lipid content, adoptive transfer of perforin- or GzmB-deficient CD8 T cells led to reduced necrotic cores, which could be attributed to reduced apoptosis of macrophages, ECs, and vascular smooth muscle cells (VSMCs). In addition to inducing caspase-dependent killing of ECs and VSMCs, GzmB can further weaken the fibrous cap by degrading collagen and other extracellular matrix proteins produced by VSMC during vascular inflammation, promoting plaque rupture and exposure of the highly thrombogenic necrotic core^27^. Exacerbation of the necrotic core can lead to restricted blood flow and mechanical stress on the fibrous cap, promoting ischemic injury and plaque rupture (Fig. 1). Taken together, these findings suggest an atherogenic role of CD8 T_EM_ in a perforin- and GzmB-dependent manner.

Clinical investigations in humans have further reinforced the atherogenic potentials of vascular-homing cytotoxic CD8 T_EM_, in which the infiltration of these cells, along with the presence of their cytotoxic molecules, carry diagnostic significance in line with disease progression. A single-cell RNA sequencing (scRNA-seq) study identified a cluster of CD8 T_EM_ whose presence in plaques increased gradually and then declined following plaque rupture. These cells were found to express high levels of cytotoxic proteins, including perforin, granulysin, and granzymes GzmA, GzmB, and GzmH^28^. Similar findings obtained by Cytometry by Time of Flight (CyTOF) analysis identified two cytotoxic T_EM_ clusters within the plaque. Notably, one cluster expressing higher levels of CD127 was enriched in asymptomatic patients who had not experienced a recent stroke or transient ischemic attack. Further analysis by Cellular Indexing of Transcriptomes and Epitopes by Sequencing (CITE-Seq) revealed decreased expression of perforin and GzmB within cells of both T_EM_ clusters in symptomatic subjects^10^. Another study of 170 patients with acute coronary syndrome demonstrated that there was enrichment of CD8 T cells within the lesions that had intact fibrous caps. In contrast, patients with ruptured fibrous caps exhibited decreased CD8 T cells within lesions, along with decreased plasma levels of granulysin, perforin, and GzmA^11^. Collectively, these results suggest that cytotoxic CD8 T_EM_ accumulate in the plaque, induce rupture, and disappear following the damage, associating morbid outcomes of atherosclerosis and the presence of T_EM_, whose cytotoxicity drives plaque rupture.

Cytotoxic T_EM_ are the most enriched CD8 T cell subset in atherosclerotic plaques compared to their abundance in the circulation^10^. Within the lesion, CD8 T_EM_ are not phenotypically homogeneous and can be broadly classified into two distinct clusters, which have been detected in several studies. These clusters are differentiated based on their association with disease progression, levels of exhaustion, tissue residency, and the differential expression of cytotoxic molecules. The first cluster (referred to as MetaCluster 20, C5 or CD8.1), represents a less exhausted, terminally differentiated cytotoxic phenotype^15,29,30^. This cluster is characterized by elevated levels of CD127, NKG7, granulysin, perforin, and GzmB, alongside reduced expression of PD-1, and the lack tissue residency markers such as CD69, CCR5, and *ITGAE*. Consistent with a migratory phenotype, this cluster also exhibits increased expression of tissue egress and vascular-homing receptors, such as S1PR2 and CX3CR1. Moreover, this is cluster preferentially located in the adjacent portion of the plaque as opposed to the necrotic core, and its presence is positively associated with disease progression. The second cluster (referred to as MetaCluster 11, C4, or CD8.0), is characterized as a tissue-resident and exhausted phenotype that lacks CD127 and expresses higher levels of PD-1 and CD69^15,25,29^. The most distinctive feature of cells in the second cluster is expression of GzmK that is positively associated with age^10^. Although GzmK+ cells are the most abundant CD8 T_EM_ phenotype in plaque, their proportional representation among all plaque CD8 T cells does not show a clear correlation with disease progression. However, depletion of CD8 T cells in aged mice, when GzmK+ cells are abundant, but not in young mice, when GzmK+ cells are rare, led to reduced atherosclerotic lesions, suggesting that the GzmK-expressing CD8 T_EM_ subset may be atherogenic^31^. Analysis of T cell clonality in mice has indicated that among CD8 T_EM_, those expressing GzmK exhibit the greatest clonal expansion in plaque, and with their exhausted state, are indicative of in situ proliferation and chronic activation.

In addition to widespread evidence supporting the atherogenic role of cytotoxic CD8 T_EM_, atheroprotective and immunomodulatory functions have also been suggested. While the killing of macrophages contributes to the formation of the necrotic core, the accumulation of macrophages alone contributes to plaque growth and inflammation. Study of human plaques has revealed an inverse relationship between macrophages and CD8 T cells, suggesting that cytotoxic CD8 T_EM_ may limit lesion expansion^25^. Additionally, the killing of activated CD4 Th1 cells in Ldlr-/- mice by cytotoxic CD8 T_EM_ has been demonstrated to limit lesion development in a Fas-FasL dependent manner^25^, and plaque antigen-activated DCs were targeted by cytotoxic CD8 T_EM_^32^. Together, this evidence indicates potential intrinsic mechanisms mediated by cytotoxic CD8 T_EM_ that modulate inflammation within atherosclerotic plaques^25,32^.

The presence of dysfunctional CD8 T cells in atherosclerotic plaques highlights the chronic inflammatory nature of ASCVD. Beyond repetitive in situ activation, the exhaustion and senescence of vascular-homing CD8 T cells can be systemically promoted by hypercholesterolemia, chronic viral infection, and autoimmune diseases. However, analyses of human plaque CD8 T cells have yet to identify a distinctly exhausted subset. Atherosclerotic plaque-derived CD8 T cells often co-express exhaustion markers such as PD-1 and senescence markers such as CD57 with cytotoxic and activation markers and are subset specific. ScRNA-seq analysis identified a subset of CD8 T cells in the core of atherosclerotic plaque that co-express transcripts for effector molecules (*IFNG, GZMA*) with exhaustion markers (*TOP2A, CTLA4, LAYN, ENTPD1*, and *HAVCR2*) and are proliferative (*MKI67*)^30^. Similarly, CyTOF analysis of plaque cytotoxic CD8 T_EM_ indicated a higher level of PD-1 expression than T_EM_ in the circulation, and reduced expression of perforin, but not GzmB, consistent with an early exhaustion phenotype. CD57 expression on CD8 T_EM_ indicates a terminally differentiated, cytotoxic, and putatively senescent phenotype, usually lacking CD28 expression. CD57 + CD28- CD8 T cells, which are expanded during chronic viral infections and with age^33,34^, exhibit a heightened state of activation, and their abundance has been correlated with incidence of atherosclerosis, particularly in people living with HIV^35,36^. Despite the low expression of Ki67, recent findings have suggested that these cells undergo expansion during atherosclerosis. It has been demonstrated that IL-6 and IL-15, cytokines that are elevated during atherosclerosis, promote the expansion of these cells, consistent with a reduced frequency of CD8 T cells expressing the IL-6 receptor α chain in patients with coronary artery disease^36,37^ (Fig. 1). IL-15 can exacerbate atherosclerosis by promoting cytotoxic capacity, CX3CR1-dependent vascular-homing, and increasing CD2 expression^15,35,38^. The co-stimulatory receptor CD2 has been implicated as a potential atherogenic co-stimulatory pathway for cells that lack CD28^38,39^. CD2 is abundantly expressed by CD57 + CD28- CD8 T_EM_, particularly in people with latent CMV infection. Its ligand, LFA-3, is highly expressed within atherosclerotic lesions, and engagement with CD2 enhances T cell receptor (TCR) activation in vitro^38^ (Fig. 1). The simultaneous expression of markers of proliferation, activation, cytotoxic, and exhaustion on CD8 T_EM_ highlights the chronic inflammatory nature of atherosclerosis. The inflammatory environment of the growing plaque may further exacerbate disease in a positive feedback manner by promoting the recruitment and expansion of cytotoxic CD57 + CD28- CD8 T_EM_.

## Antigen-specificity of vascular-homing CD8 T cells

As discussed above, CD8 T cells in plaques exhibit surface markers of memory and cellular activation compared to cells in circulation^10,14–17^. However, it is unclear whether CD8 T cells in plaques are activated and proliferate as a consequence of TCR engagement or of bystander activation via proinflammatory cytokines enriched in the atherosclerotic microenvironment, such as interferon-gamma (IFNγ), TNF, and IL-15^15^ (Fig. 1).

Various candidate antigens have been explored in human and murine atherosclerosis, particularly autoantigens stemming from dyslipidemia. Cytotoxic T_EM_ CD8 T cells specific for apolipoprotein B100 (ApoB100) have been identified and were present at higher frequencies in atherosclerosis-prone ApoE-/- mice compared to frequencies in wild-type controls that was further increased with an atherogenic diet^13^. OxLDL is another endogenous antigen that elicits adaptive immune responses^40^. OxLDL may also activate CD8 T cells in an antigen-nonspecific manner through the scavenger receptor CD36 and lectin-like oxidized low-density lipoprotein receptor-1 (LOX-1)^41,42^. Interestingly, LOX-1 exhibits structural homology with CD69, and recent investigations suggest that oxLDL binding to CD69 may have an immunomodulatory effect^43–45^. However, the specific role of this interaction concerning CD8 T cells remains unknown.

Vascular-homing CD8 T cells may also exhibit reactivity to altered self-proteins, such as those having undergone deimination, also known as citrullination, a non-reversible, inflammation-associated post-translational modification^46^. In people with RA, the presence of anti-citrullinated protein antibodies (evidence for citrullinated proteins) is associated an elevated risk of cardiovascular complications^47,48^, and citrullinated proteins have been detected within atherosclerotic plaques of patients with RA^49^. In a subset of people with RA and seropositive for anti-citrullinated protein antibodies, cytotoxic CD8 T cells that express the vascular-homing receptor CX3CR1 are activated, proliferate, and produce cytokines in response to citrullinated proteins^50^.

In addition to oxLDL, ApoB100, and altered self-proteins, viral and bacterial pathogens can also contribute to ASCVD (Fig. 1). Chronic infections like HIV and CMV increases circulating activated CD8 T cells and their presence in vascular tissues have been linked to ASCVD severity^5,15,51,52^, and acute respiratory infections often precede severe morbidities including ischemic cardiovascular disease or stroke^53^. In a recent study, Chowdhury and colleagues observed an initial increase in the TCR clonality of plaque CD8 T cells with lipid deposition and plaque maturation, followed by a substantial decrease in that clonality in fibrocalcified plaques^28^, consistent with the interpretation that there is an oligoclonal surge in T cell proliferation early in atherosclerosis followed by infiltration of polyclonal cells in late disease. They found that CD8 T cells specific for influenza, SARS-CoV-2, CMV, and Epstein-Barr virus were enriched among the T cells in advanced coronary plaques relative to their enrichment among cells in the circulation, potentially due to either expression of antigens from these viruses in the plaque or by cross-reactivity between viral antigens and endogenous vascular epitopes by molecular mimicry^28^. Additionally, CD8 T cells that are specific for bacterial antigens, such as heat shock proteins (HSPs) that are highly conserved between bacteria and humans have been identified and may be cross-reactive. HSPs can be produced by *Chlamydia pneumoniae, Porphyromonas gingivalis*, and by activated endothelial cells (ECs) in atherosclerosis^53^. CD8 T cells specific for *P. gingivalis* hsp60 have been isolated from atheroma lesions in patients with atherosclerosis^54^, and mice lacking CD8 T cells prior to infection with *C. pneumoniae* and fed a high-fat diet develop markedly reduced atherosclerosis, compared to wild-type mice under the same conditions^24^. Repletion of CD8 T cells from uninfected wild-type donor mice at the time of *C. pneumoniae* infection restores atherosclerosis development, highlighting an essential role of CD8 T cells in atherosclerosis in this model^24,53^.

## Trafficking of vascular-homing CD8 T Cells in atherosclerosis

Dynamic and tightly regulated homing mechanisms involving various chemokines and cell adhesion molecules govern the infiltration and retention of CD8 T cells within atherosclerotic lesions. Correspondingly, vascular-homing CD8 T cells display a distinct expression profile of receptors for these chemokines and cell adhesion molecules.

Chemoattraction of CD8 T cells to atherosclerotic plaques is triggered by chemokines, including CCL2, CCL3, CCL4, and CCL5, that are released from activated cells within the lesion (Fig. 1). These chemokines interact with CCR4 and CCR5 on CD8 T cells, with CCR5 expression being notably higher in plaque than in circulation^55^. In patients with acute coronary syndrome, higher CCL4 levels were associated with ruptured fibrous caps^11^. Furthermore, CCL5 levels in the blood are negatively correlated with peri-coronary artery inflammation, suggesting CCL5-mediated chemoattraction might be atheroprotective^56^. However, it remains uncertain whether this effect can be attributed to CD8 T cells, considering that CCR4, which CCL5 can also bind to, is highly expressed on immunomodulatory Th2 cells. Beyond CC chemokines, chemoattraction mediated by CXCR3 has also been implicated in atherosclerosis. Its ligands CXCL9 and CXCL10 are inflammatory chemokines induced by interferon signaling that is elevated in individuals with cardiovascular disease^57^. The chemokine CX3CL1, expressed on the surface and secreted by activated ECs, facilitates both chemoattraction and adhesion^15^. Given that CD8 T cells expressing CX3CR1 are predominantly cytotoxic, CX3CR1/CX3CL1 interactions in atherosclerosis are likely pathological.

After recruitment to the lesion site by chemokines, vascular-homing CD8 T cells initiate focal adhesion to the endothelium (Fig. 1). In our own studies, we have shown that CD57 + CD8 T cells – which are highly enriched for CX3CR1 expression – preferentially move toward, adhere to, and migrate through a vascular EC monolayer compared to cells lacking CD57, and that stimulation of the ECs with TNF increases the attraction, adhesion, and transendothelial migration of CD57 + CD8 T cells in vitro^15,38,58^. These processes are mediated by chemokines (such as CX3CL1) and cell adhesion molecules expressed on T cells and ECs, such as selectins and integrins, and are promoted by cytokines (e.g. TNF) and contact-dependent activation. Expression of *ITGB1*, *ITGA1*, and *ITGA4*, encoding integrin subunits β1, α1 and α4, respectively, are elevated in cytotoxic CD8 T cells from patients with atherosclerosis. These cells also exhibit increased adhesion to extracellular matrix substrate in vitro, compared to cytotoxic CD8 T cells from healthy controls^59^. Integrin α4 and β1 form VLA-4, the primary receptor for VCAM-1 on T cells and is essential for lymphocyte adhesion and diapedesis. In vitro adhesion of CD8 T cells to ECs cultured under atherosclerosis-mimicking hydrodynamic conditions is attenuated by blocking integrin β2 or α4 with antibodies^11^. Additionally, VCAM-1 expression on EC surface can be significantly enhanced by TNF, IL-1β, and IFNγ during atherosclerosis. However, the precise contribution of these and other mechanisms in the trafficking of vascular-homing CD8 T cells to atherosclerotic lesions remains to be fully understood, as does the kinetics of T cell infiltration throughout plaque development, and investigation of the trafficking of specific subsets in humans poses significant technical challenges.

## Therapeutics

The treatment and prevention of atherosclerosis primarily focus on managing lipid dysregulation, but targeting inflammation has become an increasingly common strategy as the role of inflammation in atherosclerosis is more widely recognized. Broad immunosuppression with colchicine has proven to be beneficial in reducing ischemic cardiovascular events in patients with recent myocardial infarction or chronic coronary artery disease^60^. Statins, which are a key atherosclerotic treatment modality, have been shown to exert immunomodulatory effects on CD8 T cell function, including downregulating co-inhibitory receptors, and altering the expression of activation, cytotoxic and exhaustion markers^61–63^. Targeting inflammatory cytokine signaling, such as for IL-1β with canakinumab, or IL-6 with tocilizumab have been suggested to be potentially beneficial^64^. The targeting of chemokine receptors has also been explored with CCR5 antagonism by maraviroc being shown to reduce atherosclerosis in both humans and mice^64^. Other strategies, such as low-dose IL-2 administration, which expands regulatory T cells, might also offer benefits in atherosclerosis^60^. Finally, atherosclerosis vaccines that employ a broad array of bacterial and lipid-related epitopes have yielded promising results in mice^65^, although CD8 T cells specific to atherosclerosis-related endogenous antigens have yet to be identified in humans, and the translatability of these mouse studies remains uncertain.

## Limitations of Current Studies

Functional studies of the role of CD8 T cells in atherosclerosis in vivo primarily utilize hyperlipidemic ApoE−/− and Ldlr−/− mice, but the development of atherosclerosis in these models is predominantly attributable to lipids. Wild-type mice do not spontaneously develop atherosclerosis and exhibit significantly fewer lesions when fed a high-fat, atherogenic diet. In contrast, ApoE−/− and Ldlr−/− mice can develop atherosclerosis even on a standard diet. Moreover, murine plaques rarely rupture, confirming that these models do not entirely replicate human atherosclerosis. In order to manipulate the CD8 T cell response, genetic knockouts are often employed which present inherent complications. For instance, MHC I knockout mice exhibit dysregulated iron homeostasis and an exaggerated innate inflammatory response^66^. Similarly, the potential for compensatory responses by other cell types following CD8 T cell depletion has not always been adequately addressed in these models. In addition, the extent of CD8 T cell depletion in tissue sites is often overlooked.

The primary limitation in human studies is that cardiovascular imaging techniques and endarterectomy specimens typically originate from advanced atherosclerosis, complicating efforts to study lesion progression. Immunophenotyping of lesional cells necessitates enzymatic digestion, with sensitive cells potentially being lost in the process. Current studies also have yet to offer a comprehensive spatial overview of the localization of CD8 T cells in atherosclerotic plaques and associated tissues or the circulation, which may play roles distinct from CD8 T cells within the plaque^67^.

## Conclusion

Plaque-infiltrating CD8 T cells play an instrumental role in atherosclerosis. Experimental mouse models of atherosclerosis have highlighted potential atherogenic and atheroprotective mechanisms for vascular-homing CD8 T cells. High-dimensional immunophenotyping of human atherosclerotic plaques has revealed considerable heterogeneity of vascular-homing CD8 T cells, with the occurrence and function relevant to various stages of disease progression. Regarding the origin of vascular-homing CD8 T cells, a considerable amount of evidence has linked chronic infections and hypercholesterolemia, which drive the clonal expansion of vascular-homing CD8 T cells peripherally and in situ. The activation and proliferation of these cells occur early in the disease onset and are chronically sustained by both systemic and local inflammation during atherosclerosis. However, the antigen-specific mechanisms and functional implications underlying the remarkable heterogeneity of vascular-homing CD8 T cells in the development and progression of atherosclerosis remain to be resolved.

## Data Availability

No datasets were generated or analyzed during the current study.
